# Quantification of deficits in lateral paw positioning after spinal cord injury in dogs

**DOI:** 10.1186/1746-6148-4-47

**Published:** 2008-11-25

**Authors:** Lindsay Hamilton, Robin JM Franklin, Nicholas D Jeffery

**Affiliations:** 1 Department of Veterinary Medicine, University of Cambridge, Madingley Road, Cambridge, CB3 0ES, UK; 2 Cambridge Centre for Brain Repair, University of Cambridge, Madingley Road, Cambridge, CB3 0ES, UK

## Abstract

**Background:**

Previous analysis of the behavioural effects of spinal cord injury has focussed on coordination in the sagittal plane of movement between joints, limb girdle pairs or thoracic and pelvic limb pairs. In this study we extend the functional analysis of the consequences of clinical thoracolumbar spinal cord injury in dogs to quantify the well-recognised deficits in lateral stability during locomotion. Dogs have a high centre of mass thereby facilitating recognition of lateral instability.

**Results:**

We confirm that errors in lateral positioning of the pelvic limb paws can be quantified and that there is a highly significant difference in variability of foot placement between normal and spinal cord injured dogs. In this study there was no detectable difference in lateral paw positioning variability between complete and incomplete injuries, but it appears that intergirdle limb coordination and appropriate lateral paw placement recover independently from one another.

**Conclusion:**

Analysis of lateral paw position in the dog provides an additional tier of analysis of outcome after spinal cord injury that will be of great value in interpreting the effects of putative therapeutic interventions.

## Background

Previous studies on recovery of pelvic limb function in spinal cord injured quadrupeds have focussed predominantly on assessing the extent to which generation of muscular activity in the pelvic limbs can produce appropriate movement and coordination of movement between pelvic and thoracic limbs in the sagittal plane [*e.g*. [1-3]]. However, spinal cord injury (SCI) also produces a loss of the ability to place the feet in the correct positions with respect to the body's centre of mass – *i.e*. the accuracy of placement in the lateral plane. This loss is implicit in many reports on pelvic limb function in experimentally-spinalised animals. For instance: the recognised need for tail support (and stimulation in some cases) to permit SCI animals to maintain walking on a treadmill belt [4,5]; the occurrence of placement errors such as limb crossing in SCI dogs with reasonable stepping ability [6]; the clinically-evident tendency for individuals with an SCI to lose their balance particularly when turning corners [7,8]. Despite recognition of its occurrence, lateral instability has rarely been quantified in animals with SCI.

Whilst mechanisms to control body posture have been studied in a range of species, vertebrate neural networks have been most thoroughly described in the lamprey. Lamprey body orientation is monitored in three dimensions by the vestibular system which mediates its effects on posture via the reticulospinal tract – the only well-developed descending pathway in this species [9-12]. However, in quadrupedal land animals maintenance of posture requires an integration of vestibular, visual and somatosensory (proprioceptive) sensory inputs. The relative significance of these inputs differs between body regions; input from the vestibular system being relatively more important for maintaining head orientation and thoracic limb stability, and somatosensory information from the limbs being more important for maintaining trunk and pelvic limb stability [13-16]. Additionally, experiments on decerebrate cats show that neural structures required for maintenance of body posture are located in the cerebellum, brain stem and spinal cord, since such animals are able to engage righting reflexes to correct their posture when abnormally positioned. Two brainstem areas important in control of postural muscle tone are the dorsal and ventral tegmental fields, with integration between the output from these areas and other descending locomotor signals occurring in the medullary reticular formation and spinal cord [17,18].

In previous studies the ability of quadrupeds to control limb position relative to the trunk has been studied mainly by examining the ability of an animal to maintain balance during adaption to perturbation of a static posture, usually by means of a tilting platform [19-21]. Although spinalised animals are able to maintain a standing posture (for very limited periods) they have profoundly impaired ability to respond to external perturbation of their posture. This is thought to result from lack of recruitment of flexor muscle activity for this particular purpose, which is dependent on supraspinal control mechanisms [20]. Investigations on spinalised rabbits further suggest that ventral lesions are associated with more pronounced and lasting deficit in postural correction than either dorsal or lateral lesions [22].

During locomotion, anticipatory adaptations in posture are made using more complex control than that required for making the reflex, balance-maintaining corrective responses to changes in static body posture described above. Pre-emptive responses are needed when either internal or external factors affecting posture are expected, most simply between each step cycle during steady locomotion. Further control is needed for maintaining balance through changes in speed, direction or terrain. Abnormalities such as increased lateral trunk sway, a wide-based stance with the trunk angled to one side, and frequent stumbling have been described during treadmill locomotion in spinalised cats, all of which were exacerbated by inclining the treadmill or increasing its speed [5]. However these observations are subjective, implying the need to develop methods to quantify lateral instability. In a recent study, Ichiyama et al (2008) [23], chose the parameter of 'width of pelvic limb stance' as a means of examining paw placement in the lateral plane in their analysis of the effects of locomotor training, epidural stimulation and intraperitoneal quipazine on the gait patterns of rats after complete thoracolumbar cord transection. Whilst this parameter provides valuable information on paw placement, especially in a homogenous group of laboratory rats, our previous observations had suggested the need to examine *variability *in lateral placement as well as absolute values of interpaw distance.

Therefore in this current study we wished to extend our previous quantification of coordination of thoracic and pelvic limb in the sagittal plane to examine the variability (or 'consistency') of pelvic limb placement in the lateral plane (here termed the 'y-plane') in dogs that had suffered thoracolumbar spinal cord injury. Therefore we used the *coefficient of variation *as a unitless parameter to summarise the amount of variability in the lateral positioning of the limbs of each girdle. This descriptive parameter has frequently been used to describe and analyse aspects of gait variability in humans [24], notably to assess the risk of falls in elderly people [25].

Clinically, in both humans [26] and dogs [27], spinal cord injury (SCI) can be divided into 'complete' – in which there is no discernible transmission of impulses across the damaged region of the spinal cord – and 'incomplete', injuries. Previously, we demonstrated loss of coordination between thoracic and pelvic sagittal limb movements in dogs that had incurred clinically complete lesions [28]. However, it is possible that recovery of intergirdle limb coordination in the sagittal plane could be mediated by propriospinal pathways rather than requiring instruction from motor centres in the brain. We reasoned that, since control of lateral stability is dependent upon brainstem centres (see above), it should be possible to define two different types of coordination of pelvic limb placement after thoracolumbar SCI: that defined by thoracic-pelvic limb coordination in the sagittal ('x') plane and that defined by the positioning of the pelvic limbs in relation to the trunk in the lateral ('y') plane (*i.e*. mediated by extrinsic pathways between the spinal cord and brainstem).

Therefore in this study we analysed stepping after clinical thoracolumbar SCI in dogs to test the hypothesis that placement of the pelvic limbs in the lateral plane would be abnormally variable in all dogs with complete SCI but could be normal in those with incomplete SCI, depending on the severity of the lesion.

## Results

### Normal dogs

**Figure 1:**
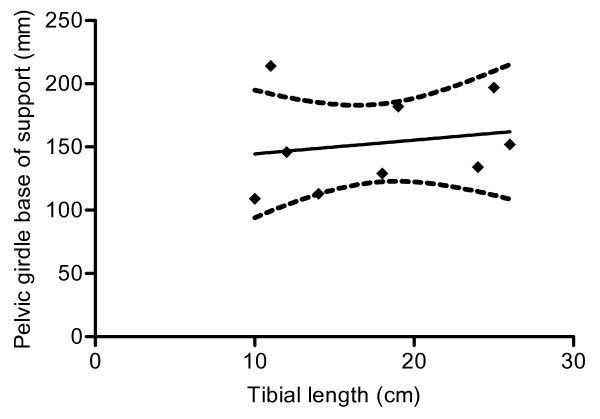
**XY plot to show relationship between tibial length and pelvic girdle base of support in normal dogs solid line shows imposed linear regression line and dashed lines indicate 95% confidence intervals**. There is not a significant correlation between these variables (r = 0.33; p = 0.39).

There was considerable variability in the width of support provided by the thoracic (87–179 mm) and pelvic (109–213 mm) limb pairs, reflecting the conformational heterogeneity in the tested sample of the domestic dog population. Furthermore, there was considerable variability in the relative width of support between thoracic and pelvic limb girdles – *i.e*. some animals had wider base of support in the thoracic limbs and some in the pelvic limbs. We examined the possibility of a correlation between physical size and the width of support in the pelvic limbs, using the length of the tibia as a means of estimating the size of each dog. There was no significant correlation (Spearman's test r = 0.33; p = 0.39; see Fig 1), reflecting the high diversity of dog conformation.

Next we quantified the variability in foot placement by calculating the *coefficient of variation *(CV: standard deviation divided by the mean, see methods) for the thoracic and pelvic limbs pairs of each dog. This calculated ratio revealed differences in the variability of limb placement between individual dogs that were of similar magnitude in thoracic (0.08–0.27) and pelvic (0.08–0.17) limbs. Since we were interested in ultimately comparing the reliability of pelvic limb placement with that in the thoracic limbs we then divided the *coefficient of variation *for the pelvic limbs into that for the thoracic limbs, producing a figure to define the difference in foot placement variability between limbs in the two girdles. As would be expected in normal individuals, these fell within a narrow range (0.86–2.5), with a mean value close to 1 (1.29).

We then examined the effect of variation in treadmill speed on these parameters. In neither the thoracic (paired t test; p = 0.58), nor the pelvic (paired t test; p = 0.26) limbs was there an effect of speed on width of support. As expected, the ratio between thoracic and pelvic limb variability was also unchanged by altering the treadmill speed (paired t test; p = 0.36).

### SCI dogs

#### a) Incomplete SCI

Incomplete SCI resulted from a variety of causes in dogs included in this study; these are listed in Table 1. The thoracic limb base of support varied widely within this group (82–241 mm), which is similar to the range in normal dogs. However, the base of support through the pelvic limbs (45–260 mm) was far more variable than that found in normal animals. Nevertheless, the *median *values for width of base support were similar in this group to those in the normal group (thoracic limbs: Kruskal-Wallis test, p = 0.71; pelvic limbs: Kruskal-Wallis test, p = 0.17) – perhaps reflecting the anatomical constraints on possible positions of the paw in the y-plane. There was no significant correlation between tibial length and the width of support in the pelvic limbs (Spearman's test, p = 0.35; p < 0.05).

**Table 1:** Causes of spinal cord injury in tested animals

**Cause of complete injury**	**Cause of incomplete injury**
L1/2 IVD	T11 caudal epiphyseal fracture
T13/L1 IVD	T13/L1 nephroblastoma
T12/13 IVD	T12/13 IVD
T12/13 fracture-luxation	L1/2 IVD
T13/L1 IVD	L1/2 IVD
T13/L1 IVD	L1/2 IVD
T12/13 subluxation	T8 hemivertebra
	T13/L1 IVD
	L1/2 IVD
	T12/13 IVD
	T13/L1 IVD
	T13/L1 IVD
	L1/2 IVD

**Figure 2:**
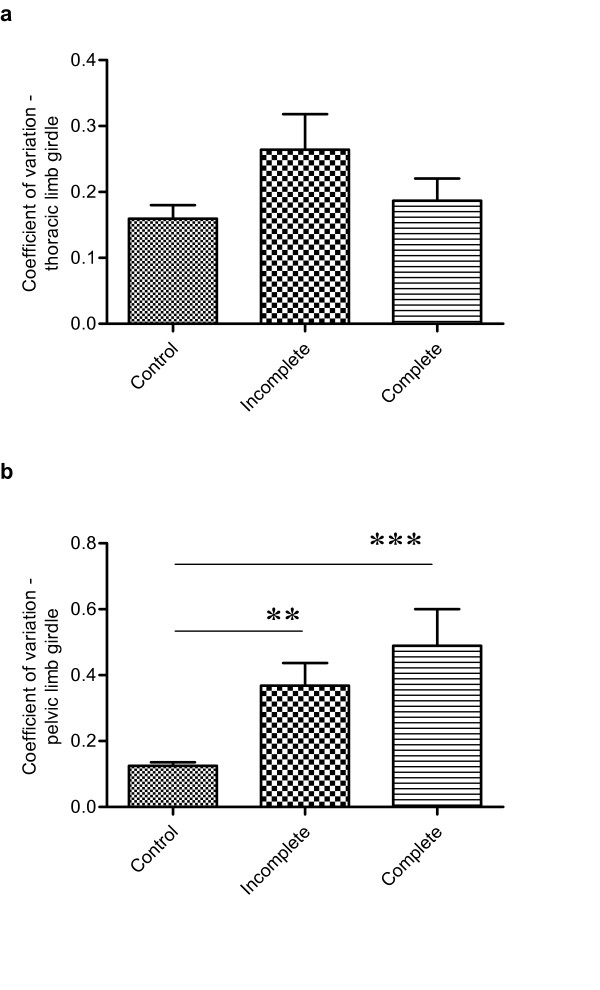
**Bar charts to illustrate the variability of paw placement position in the lateral ('y') plane**. Variability has been quantified by dividing the mean distance between the limb pairs by the standard deviation, to produce the coefficient of variation.a. Variability in thoracic paw placement does not vary amongst the groups (Kruskal-Wallis test p = 0.27). b. There is increased inconsistency in placement of pelvic paws after both incomplete (** p < 0.01; Dunn's post hoc test) and complete (*** p < 0.001; Dunn's test) thoracolumbar spinal cord injury, when compared with normal dogs (Kruskal-Wallis test p < 0.0001).

**Figure 3:**
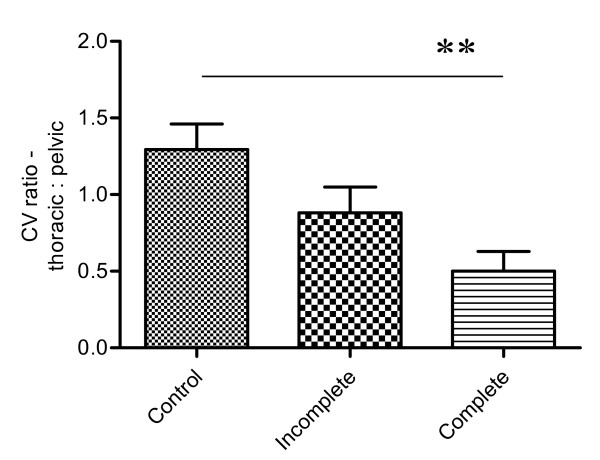
**Bar chart to illustrate the relationship between variability of thoracic and pelvic paw placement in the different groups of dogs (Kruskal-Wallis test p = 0.0069)**. There is a significant difference in this parameter between normal dogs and those with complete (** p < 0.01; Dunn's test) thoracolumbar spinal cord injury.

Next we examined the variability in limb placement by calculating the *coefficient of variation *as described above. Again, there were differences in this parameter between individual animals in both thoracic (0.13–0.79) and pelvic (0.16–1.02) limbs, including some notably much larger values than those calculated for normal individuals, implying an increased variability of foot placement. The calculated CV were used to compare consistency of foot placement compared amongst all groups of dogs. This demonstrated that thoracic foot placement was not significantly different between groups (Kruskal-Wallis test, p = 0.27), but was significantly different for the pelvic limbs (Kruskal-Wallis test; p < 0.0001) including a significantly reduced consistency of placement between normal and incomplete injury cases (Dunn's *post hoc *test; p < 0.05, and see Fig 2). We considered the possibility that this result might have been a consequence of different limb lengths in this group but analysis showed that all tested groups of dog had statistically similar tibial lengths (Kruskal-Wallis test, p = 0.13 and see Table 2). These results confirmed that there was a significantly greater inconsistency in pelvic limb placement after incomplete SCI, although the ratio of thoracic and pelvic coefficient of variation was not significantly different from that of normal animals (Fig 3).

**Table 2:** Tibial lengths in the three tested groups

**Normal**	**Incomplete**	**Complete**
11	15	8
12	11	8
19	20	15
26	19	6.5
24	16	14
10	7	7
25	8	16
18	9	
14	13	
	22	
	6.5	
	13	
	7	

Tibial lengths are statistically equal amongst the three groups (Kruskal-Wallis test p = 0.13).

This group of dogs had incomplete injuries and their locomotor capability changed with time. Using the mean diagonal coupling interval parameter we described previously [27] we could divide these dogs into sub-groups with either normal or abnormal coordination in the sagittal ('x') plane and ask the question whether there was a concomitant alteration in the variability of lateral foot placement during recovery of locomotion. There was some change in the *coefficient of variability *of pelvic limb placement at different stages of recovery but this was not statistically significant (paired t test, p = 0.1), implying that there was no major alteration in the ability to place the limbs correctly in the lateral ('y') plane during recovery of intergirdle coordination. At the time at which these dogs exhibited normal x-plane coordination only two of eight animals exhibited variability of lateral foot placement within the range of the normal dogs.

#### b) Complete SCI

The causes of complete SCI of dogs included in this study are listed in Table 1. Base of support values in both the thoracic (66.4–215.5 mm) and pelvic (71.9–218.1 mm) limbs were variable, as in the other two groups. Similarly, there was variability in the reliability of foot placement in both the thoracic and pelvic limb girdles: i) in the thoracic limbs the variability ratio varied between 0.06 and 0.31 – which was not significantly different from that found in either normal or incomplete SCI cases (Kruskal-Wallis test); ii) in the pelvic limbs the *coefficient of variation *ranged from 0.25 to 1.1 and was significantly different from that in normal dogs (Kruskal-Wallis test; p < 0.0001, Dunn's *post hoc *test; p < 0.05), but not from those with incomplete injuries (see Fig 2). Similar results were obtained when examining the relationship between the *coefficient of variation *of the thoracic and pelvic girdles; animals with complete SCI had a significantly different ratio from the normal animals (Kruskal-Wallis test p = 0.0069; Dunn's *post hoc *test p < 0.05, see Fig 3) but not from those with incomplete injuries.

### Analysis of use of support band

Both groups of SCI dogs could not walk unsupported on the treadmill, which may have had an effect on the degree to which lateral movements were permitted. Therefore we also examined the effect of band support on the normal dogs, in case there was an effect to exacerbate the increased variability of weight support in the SCI dogs. When examining normal dogs the band did not alter stepping inconsistency, as measured by the variability ratio in either the thoracic (p = 0.22; paired t test) or the pelvic (p = 0.33; paired t test) limbs.

## Discussion

The data presented here quantify for the first time the deficits in lateral foot placement associated with clinical spinal cord injury in dogs and confirm that there is excessive inconsistency in positioning, accounting for lateral instability during locomotion. Whilst normal dogs have very limited variability in lateral foot placement during locomotion this is markedly increased after both complete and incomplete SCI. This quantification is valuable since it provides an additional outcome measure by which to analyse the effects of therapeutic interventions. Moreover, it is extremely robust, since it is likely that our methods include an inevitable underestimate of the lack of coordination in the lateral plane, because provision of an abdominal band support is essential to allow data to be collected at all, but inherently limits variability in lateral paw placement. Furthermore, variability does not appear to be affected by changes in locomotor speed.

Our data show that although it is possible for animals with clinically 'complete' thoracolumbar SCI (*i.e*. with no discernible sensory transmission to the head across the lesion) to generate stepping movements they do not recover the ability to correctly place the feet in the lateral plane. By comparison, after complete SCI untrained rats (but treated with epidural stimulation and quipazine) had a similar pelvic limb stance width to unlesioned animals [23]; however, that study did not examine *variability *of paw placement in the lateral plane. Surprisingly, in our data there appeared not to be a difference in variability in lateral paw placement between complete and incomplete SCI suggesting that both brainstem-spinal cord and intra-cord connectivity are disrupted to a similar degree between these two groups – at least at the stage of recovery at which the incomplete SCI animals were examined. However, our data show that recovery of intergirdle limb coordination can occur in the absence of correct placement in the lateral plane. This suggests that either: i) different pathways (as consistent with the previous experimental data cited in the Introduction) or, ii) a different degree of pathway integrity, is required for these two different forms of coordination. None of the dogs without normal intergirdle coordination placed the pelvic limbs correctly in the lateral plane. The converse question – whether animals that recover lateral stability always have normal coordination in the x-plane – cannot be answered here, since only one SCI dog had lateral plane movements within normal limits (and also had normal x-plane coordination). It is possible that lateral plane coordination represents a more advanced stage of recovery, which is dependent on brainstem-spinal cord connections and cannot occur in animals with very severe SCI.

Our findings confirm that two components of limb coordination – lateral stability and intergirdle limb coordination – can be individually analysed during recovery, or after interventions designed to enhance functional outcome. Recognition of, and the ability to quantify, these different components of recovery of limb control after SCI provides a mechanism for increased sophistication of analysis of outcome of interventions designed to promote recovery after severe SCI. A recent study has shown that, in rats, pelvic limb stepping can be mediated solely through propriospinal networks caudal to a spinal cord lesion [29], which suggests that evaluation of potentially useful interventions after SCI should also focus on features of the gait apart from simple stepping. Moreover, for human patients to derive maximum benefit from an intervention after SCI it is usually assumed that there would need to be control mediated from the brain – in fact, most usefully from the cerebral cortex (see [30]). Although our gait analysis would not allow us to determine whether corticospinal connections can be restored by an experimental intervention, analysis of limb movements in the lateral plane provides an evaluation of brainstem-spinal connectivity and, we show here, can be quantified in dogs.

These findings also highlight the value of SCI dogs in providing an enhanced analysis of the effect of interventions designed to improve the functional outcome after severe SCI, since limb movements in both the sagittal and lateral planes can be examined. In contrast to rodents, dogs have a relatively high centre of mass, which facilitates analysis of limb movement in directions other than the sagittal plane, with the implication that it would be useful to analyse in clinical dog SCI patients the effects of interventions that have previously proven to be of value in experimental rodent models.

## Conclusion

We show here that it is possible to quantify the increased variability in lateral support that results from thoracolumbar SCI and that this may recover independently of limb girdle coordination in the sagittal plane. This ability to analyse locomotor outcome in two planes (sagittal and lateral) will permit greater sophistication in measurement of the effects of interventions designed to improve locomotor function.

## Methods

Dogs in this study were presented to the Dept Veterinary Medicine for treatment of clinical SCI, or were owned by members of staff (normal dogs). The study was carried out in the UK under the jurisdiction of the Veterinary Surgeons Act (1966).

### Normal dogs (n = 9)

These were of a variety of breeds and types, and owned by members of staff in the Department.

### Spinal cord injured dogs (n = 20)

We restricted this investigation to dogs that had incurred thoracolumbar SCI, but exhibited perceptible stepping movements in the pelvic limbs, the clinical details are included in Table 1. Stepping activity was precisely defined by using data derived from digital gait data on step length; only dogs that exhibited pelvic limb steps of at least 40% of the length of the thoracic limbs were examined (in normal dogs pelvic limb steps vary between 80–109% of thoracic limb step length [data on file]).

SCI dogs were divided into two sub-groups:

#### a) 'Incomplete' SCI (n = 13)

These dogs had sustained thoracolumbar SCI but had not lost sensory function to the paws of the pelvic limbs. The term 'incomplete' is used here to facilitate comparison with human SCI patients and this group of dogs was categorised as having injuries equivalent to ASIA grade D [26]. These dogs had sustained SCI sufficiently severe to prevent them from walking unaided but not to lose conscious pain perception in the pelvic limbs and had undergone conventional clinical treatment (decompressive spinal surgery or cage rest and physical therapy) and subsequently recovered the ability to step on the treadmill with support. These dogs were examined at a stage of recovery at which stepping occurred, although they were unable to walk without support of the hindquarters (and therefore exhibited comparable stepping competence to their counterparts that had 'complete' SCI – see below).

To permit comparisons of reliability of foot placement in the lateral plane at different stages of recovery after incomplete SCI we further sub-divided data on this cohort of dogs into two groups according to the extent of coordination between the thoracic and pelvic limbs. Thus, we compared the variability of foot placement in the y plane at the stage at which we could define intergirdle coordination as poor (*i.e*. they had a 'mean diagonal coupling interval' [see [28]] of greater than 0.1) with that at which they had recovered normal intergirdle limb coordination (*i.e*. mean diagonal coupling interval of less than 0.1).

#### b) 'Complete' SCI (n = 7)

These dogs had chronic SCI (onset was at least six months previously) with absent sensory function (including absent conscious pain perception) and absent voluntary movements in the pelvic limbs; these dogs therefore were equivalent to humans with ASIA grade A injuries. Despite the chronic severe SCI, all dogs in this category were able to make pelvic limb stepping movements when partially supported on the treadmill (this effect is sometimes referred to as 'spinal stepping' – see [6]) and they fulfilled the criteria of exhibiting pelvic limb step lengths of at least 40% of that of the thoracic limbs.

### Treadmill stepping

The methods of acquiring the digital gait data have been described previously [28]. Briefly, dogs were held by a leash around the neck and encouraged to walk on the treadmill, until they found a comfortable speed (varying from 1–3 kmh^-1^) at which to walk. SCI dogs required support in the form of a support band placed under the abdomen to prevent falling during locomotion. Tension in the support band was adjusted so that the thoracolumbar part of the vertebral column was parallel to the treadmill belt surface and minor adjustments were made to maximise the intensity of stepping movements. Since ~60% of body weight is supported through the thoracic limbs of normal dogs and there is variation from instant to instant in the precise loading of the limbs during acceleration and deceleration [31] we estimate the load bearing of the band to be between ~10 and 35% of body weight during a step cycle, depending on the degree of lateral sway.

### Equipment set-up

Four infrared motion capture cameras with a recording frequency of 100 Hz (Qualisys, Sweden) were positioned around a standard ex-gymnasium treadmill and calibrated to permit recording from the entire belt surface. The lateral plane was designated as the 'y-plane' in these recordings. 10 mm reflective markers were attached to the skin overlying specific anatomical landmarks: lateral fifth phalange, lateral humeral epicondyle, ulnar styloid process, greater trochanter of the femur, lateral femoral epicondyle, lateral malleolus of the tibia, and the interscapular region dorsal to vertebra C7.

### Recording

During recording, the treadmill speed was set to the speed determined previously, at which the dog was walking consistently, and 60 seconds of motion was recorded. The treadmill speed was then increased and recordings repeated at a variety of speeds. In some normal animals we examined the effect of applying belt support, to act as a control for this means of assistance that was essential for dogs that had spinal cord injuries.

### Gait analysis

Processing of the recorded images was initially carried out using Qualisys Track Manager software (Qualisys QTM, Sweden). The 15 individual markers were identified and labelled to construct a 3D stick-diagram representation of the dog. Visual examination of lateral and forward movement was displayed in QTM graphical plots of y- (lateral) and x- (sagittal) plane position and used to exclude sections of data in which a dog was not walking consistently (recognised by abrupt irregularity in the plot, indicating acceleration or deceleration, or sudden movement to one side). Subsequent analysis of coordination was focussed on data obtained from the paws only (phalangeal markers). Positional data from y-plane plots of each paw were exported from QTM into Matlab (Release 14, Student version) and the distance between consecutive paw placements (*i.e*. from left to right and *vice versa*) of both limb girdles was calculated using a custom-designed script. For each paw, the instant of each consecutive paw placement was identified from the maximal stationary values in the sagittal (x-plane) data (corresponding to the maximal extent of paw protraction immediately prior to its placement on the treadmill surface); the y-plane coordinates of the paw at that instant were then extracted by the Matlab script and exported into an Excel file (and see Additional File 1).

Initially therefore we determined the *base of support *for the thoracic and pelvic limb girdles respectively. Because of the variability in conformation of our study group of dogs we anticipated the need to apply a formula to determine normal ratios for the base of weight support in the thoracic and pelvic limb girdles. However, more importantly, in this study we were primarily interested in the degree of *variability *in foot placement, rather than the mean position (since that would be restricted by the anatomy of the pelvic limb). We reasoned that animals that exhibited greater unreliability in placement would have an abnormally wide distribution of values compared to the mean. Therefore we calculated the *coefficient of variation *in foot placement by using the base of support data and dividing the standard deviation by the mean for the thoracic and pelvic limb pairs individually [32]. We then compared this ratio between groups of dogs. Because we were interested in whether there was a difference in inconsistency of foot placement between the pelvic and thoracic limb girdles we then derived a second ratio – between the coefficient of variation of the pelvic and thoracic limb girdles. This parameter was then also compared amongst the groups.

### Estimation of limb length

Measurement of 'limb length' in dogs is not straightforward, since different conformations of dog will stand with the joints at different angles, therefore we measured tibial length as a surrogate that would accurately represent the range in size of dog in each group. These measurements were made from the digital data acquired through the QTM software indicating the relative locations of the stifle (knee) and hock (ankle) joints. Tibial lengths for dogs in each group are listed in Table 2.

### Statistical analysis

Data acquired in QTM were transferred as numerical data into Matlab. A custom-written script was used to extract the data points of interest and standard matrix addition or subtraction was used to calculate time intervals and position as described above. The resulting data was assembled in Excel spreadsheets and transferred into GraphPad Prism (Version 5.0 for Windows) for statistical analysis.

For each animal there were columns of data listing the distance between the intragirdle paw pairs at placement on the treadmill. From these we calculated the means, standard deviations and coefficients of variation for comparison between different groups. All groups of data (normal, incomplete and complete SCI) were initially compared using the Kruskal-Wallis test, followed by *post hoc *Dunn's tests where appropriate to determine differences between specific groups if significance was detected in the Kruskal-Wallis test. Where this occurred we have reported results of *post hoc *tests, full details are given in figure legends. Paired Student's t tests were used to compare data derived from normal animals at different speeds and walking with and without abdominal band support. The Mann-Whitney test was used to compare data from normal and lame dogs. For all tests, significance was assumed when p < 0.05.

## Authors' contributions

LH carried out the locomotor testing and wrote part of the manuscript. RJMF aided in experimental design, provision of laboratory facilities and editing of the manuscript. NDJ designed the study, wrote most of the manuscript and carried out the statistical analysis.

## Supplementary Material

Additional file 1 **Data processing example**. This additional file provides information on the method used to transform the raw data into the summary description and statistical analysis of lateral foot positioning. Click here for file
