# Supplementary material for: Quantification of deficits in lateral paw positioning after spinal cord injury in dogs
Source: BMC Vet Res. 2008 Nov 25;4:47. doi: 10.1186/1746-6148-4-47 (PMC2631515; doi:10.1186/1746-6148-4-47)
Supplement: Additional file 1 — Data processing example. This additional file provides information on the method used to transform the raw data into the summary description and statistical analysis of lateral foot positioning. [file 1746-6148-4-47-S1.doc]

**Additional file 1**

***Data processing example***

We used the lateral distance between limb girdle pairs as the basic measurement, but this distance cannot be measured simply in an animal that is moving. Therefore we measured the lateral distance between one paw placement (say the left pelvic limb paw) and the subsequent placement of its partner (the right pelvic limb paw). To do this we needed to identify the instant when each paw was placed onto the treadmill surface, which can be done on the sagittal (x-plane) data – since this moment is the maximum forward motion of the paw during each step cycle. The Matlab script was used to automatically identify these instants and then extract the y-plane coordinates of each paw when it contacted the treadmill.

The data on y-plane position was then extracted from Matlab and transformed into an Excel file (see Additional File Table 1 below). Subsequent analysis was done through routine Excel functions. Graphs of the calculated data (such as coefficients of variation) were made in GraphPad Prism (Version 5 for Windows).

***Additional file Table 1***

| *RH paw* | *R Elbow* | *LH paw* | *L Elbow* | ***Elbows*** | ***HL paws*** |
| --- | --- | --- | --- | --- | --- |
| 64.51 | -14.34 | 219.05 | 204.39 | **218.73** | **154.54** |
| 64.15 | -14.7 | 223.09 | 203.57 | **218.27** | **158.94** |
| 64.19 | -15.04 | 225.52 | 202.65 | **217.69** | **161.33** |
| 63.78 | -15.19 | 226.45 | 201.59 | **216.78** | **162.67** |
| 63.44 | -15.6 | 226 | 200.22 | **215.82** | **162.56** |
| 46.46 | -16.28 | 224.24 | 199.02 | **215.3** | **177.78** |
| 41.89 | -17.16 | 221.58 | 197.84 | **215** | **179.69** |
| 62.88 | -17.88 | 217.58 | 196.56 | **214.44** | **154.7** |
| 62.39 | -18.61 | 212.76 | 195.51 | **214.12** | **150.37** |
| 62.45 | -19.52 | 207.47 | 194.76 | **214.28** | **145.02** |
